# Cocoa flavanol supplementation in optimizing post-exercise glycemic control in rats with normoglycemia or diabetes mellitus: findings from the ECODIA study

**DOI:** 10.20945/2359-4292-2024-0169

**Published:** 2025-03-24

**Authors:** Bruno Pereira Melo, Joyce Camilla Cruz de Oliveira, Aline Cruz Zacarias, Letícia Maria de Souza Cordeiro, João Gabriel da Silveira Rodrigues, Mara Lívia dos Santos, Gleide Fernandes de Avelar, Romain Meeusen, Elsa Heyman, Camila Berbert Gomes, Pedro Henrique Madureira Ogando, Danusa Dias Soares

**Affiliations:** 1 Universidade Federal de Minas Gerais, Departamento de Educação Física, Laboratório de Fisiologia do Exercício, Belo Horizonte, MG, Brasil; 2 Centro Universitário ITOP (UNITOP) - Instituto Tocantinense de Ensino Superior e Pesquisa Ltda., Palmas, TO, Brasil; 3 ImaginAb, Inglewood, CA, USA; 4 Universidade Federal de Minas Gerais, Departamento de Morfologia, Laboratório de Biologia Celular, Belo Horizonte, MG, Brasil; 5 Human Physiology Research Group, Faculty of Physical Education and Physical Therapy, Vrije Universiteit Brussel, Brussels, Belgium; 6 Universidade Federal de Minas Gerais, Departamento de Morfologia, Laboratório de Biologia Celular, Belo Horizonte, MG, Brasil; 7 Institut Universitaire de France, Paris, France

**Keywords:** Diabetes, polyphenols, glucose, physical exercise, obesity

## Abstract

**Objective:**

This study investigated the acute effects of cocoa flavanol (CF) supplementation on glucose homeostasis, aerobic performance, and lactate concentration in rats with type 1 diabetes mellitus (T1DM), type 2 diabetes mellitus (T2DM), and normoglycemia (NORM).

**Materials and methods:**

The study included 28 male Wistar rats (220-290 g). Induction of T1DM (n = 8) was achieved through intraperitoneal injection of streptozotocin, while T2DM (n = 10) was induced using an *ad libitum* high-fat diet combined with a fructose-rich beverage. The rats in the NORM group (n = 10) received a standard diet for 30 days. Two experiments were conducted: (^1^) T1DM rats performed two successive 30-minute treadmill runs below the anaerobic threshold and (^2^) T2DM and NORM rats underwent two incremental maximal treadmill running tests, both after CF or placebo supplementation. Blood glucose concentrations were measured from pre-exercise to 60 minutes post-exercise.

**Results:**

Glycemic reduction at 60 minutes post-exercise was significantly potentiated by CF compared with placebo supplementation in T1DM, T2DM, and normoglycemic rats (p < 0.05 for all). In T2DM rats, CF induced a glycemic response comparable to the NORM placebo-supplemented condition. These effects of CF persisted despite variations in aerobic performance or lactate concentration after incremental exercise.

**Conclusion:**

Supplementation with CF prior to physical exercise elicited a pronounced post-aerobic exercise glycemic reduction. This represents a promising strategy for mitigating the duration of hyperglycemia exposure after physical exercise.

## INTRODUCTION

By the end of this decade, diabetes mellitus will affect an estimated 578 million individuals worldwide (^1^). This chronic condition encompasses four types but is represented predominantly by type 1 diabetes mellitus (T1DM) and type 2 diabetes mellitus (T2DM), which collectively account for over 95% of the cases. According to the American Diabetes Association (^2^), T1DM results from autoimmune destruction of beta cells, leading to absolute insulin deficiency, while T2DM is characterized by insulin resistance and metabolic syndrome resulting in a progressive loss of insulin secretion by beta cells (^2,3,4^). Both T1DM and T2DM manifest clinically with hyperglycemia, placing individuals at risk of developing common chronic complications once this occurs (^2^). Therefore, maintaining optimal glycemic control is a cornerstone of the successful treatment of diabetes mellitus.

Physical exercise is a non-pharmacological strategy that has proven effective in optimizing glycemic control and conferring additional benefits (*e.g.,* enhanced cardiorespiratory fitness) in both T2DM (^3^) and T1DM (^4^). A single aerobic exercise session induces immediate and sustained reductions in glycemia, influencing subsequent daily mean glycemic levels (^5^). Repeated exercise sessions may further decrease postprandial glycemia (^5^), thereby improving overall glycemic control (^6^). Given that the duration of hyperglycemia exposure significantly contributes to metabolic complications in diabetes, strategies to mitigate or reduce this exposure have received considerable attention. Factors influencing glycemic reductions induced by exercise include the intensity of aerobic exercise, the total work produced during exercise, drug therapy, and diet (^7^).

Bioactive compounds, particularly polyphenols or phenolic compounds, have emerged as crucial components in diets linked to health and disease prevention. Polyphenols exhibit positive effects on health (^8,9^). They are divided into tannins (polymeric polyphenols) and flavonoids. Flavanols, which are polyphenols abundant in cocoa, have been associated with various cardiometabolic benefits (^9,10^), including improvements in glycemic metabolism (^11^), vasoreactivity (^12^), and endothelial function (^13^), resulting in a decreased risk of cardiovascular disease and insulin resistance (^14,15^). Several phenolic compounds are present in cocoa, *e.g.,* flavanols, anthocyanins, flavones, and flavanones. In flavanols, (+)-catechin and (-)-epicatechin are the predominant bioactive monomers (^8^).

Cocoa flavanols (CF) have been shown to activate signaling pathways involved in glucose uptake in peripheral tissues (^10^) and enhance oxidative phosphorylation and aerobic capacity (^11,16^). Experimental studies in Zucker diabetic fat rats have shown that CF supplementation over 9 weeks reduces hyperglycemia, improves insulin sensitivity, and increases beta-cell mass and function (^16^). Chronic CF supplementation also promotes antidiabetic effects by reducing insulin resistance, inflammation, and oxidative stress in muscle and by promoting GLUT-4 translocation via PI3K/AKT and AMPK pathways (^10^).

Although the molecular mechanisms of glucose metabolism following chronic CF supplementation have been explored, the effects of acute CF doses on glycemic improvement after exercise and on physical performance under different glucometabolic conditions remain unknown. Therefore, we aimed to investigate in this study the acute effects of CF supplementation on short-term glucose recovery after aerobic exercise in insulin-deficient (T1DM), insulin-resistant (T2DM), and normoglycemic (NORM) rats. Additionally, we explored the impact of acute CF supplementation on aerobic performance and lactate concentration in T2DM rats. We hypothesized that acute CF supplementation would attenuate the hyperglycemic status following aerobic exercise.

## MATERIALS AND METHODS

### Animal and ethical care

The present study was conducted in accordance with the Brazilian National Council norms for the Control of Animal Experimentation after receiving approval from the University Ethics Committee (CEUA-UFMG, protocols 109/2016 and 110/2016).

Twenty-eight Wistar rats (220-290 g, approximately 10 weeks old) were sourced from the vivarium at the Institute of Biological Sciences, *Universidade Federal de Minas Gerais.* The rats were housed in collective cages (four animals per cage) under controlled conditions (temperature of 24 ± 1 °C, light from 7 am to 7 pm) and acclimatized for 5 days in the animal research facility of the Physiology Exercise Laboratory.

### Experimental design (first experiment)

Induction of T1DM was accomplished with a single intraperitoneal injection (65 mg/kg body mass) of streptozotocin (Sigma Aldrich, St. Louis, MO, USA) dissolved in sodium citrate buffer 0.1 M (pH 4.5) (^17^). Three days after the streptozotocin injection, T1DM was confirmed by the occurrence of polydipsia, polyuria, and hyperglycemia (above 300 mg/dL) (^17^). All animals used in this experiment were confirmed to be hyperglycemic (12-hour fasting glucose = 300 mg/dL) after T1DM induction. Insulin therapy commenced with 2 IU of NPH insulin twice daily (Humulin NPH, Eli Lilly do Brasil Ltda., São Paulo, SP, Brazil), and the morning dose was omitted on experiment days.

After T1DM induction, the rats (265.2 ± 8.4 g of body weight; n = 8) underwent three randomized experimental conditions: two moderate-intensity aerobic exercise sessions after receiving either CF (T1DM.CF) or placebo (T1DM.PLA) supplementation and a non-exercise, non-supplementation condition (T1DM.CON). These sessions were spaced by at least 72 hours of rest during the washout period. The supplementation (CF or placebo) was administered to the animals 1 hour before each exercise session. This timing of CF supplementation was based on preliminary studies. Both bioactive CF monomers (-)-epicatechin and (+)-catechin are associated with several health benefits (^16^). Specifically, (-)-epicatechin is absorbed in the digestive tract of rats, reaching maximum plasma concentrations 30-60 minutes after oral gavage (^18,19^). Therefore, the physical exercise sessions were performed at the peak of bioactive CF monomers in the bloodstream.

Ten capillary blood glucose concentrations were measured at various intervals (pre-gavage, pre-exercise, immediately after exercise, and at 15, 30, and 60 minutes after exercise). After oral gavage and exercise, the rats were placed in collective cages without access to water or food for 2 hours.

#### Exercise protocol and control condition

Before the exercise sessions, T1DM rats underwent 5 days of familiarization, which comprised 10 minutes of progressively intense running on a treadmill (1015 m/min). In these familiarization sessions, the treadmill inclination was fixed at 5%, and an electrical stimulus, set at 0.2 mA, was applied (^20^). The exercise sessions consisted of a 30-minute run at 18 m/min on a treadmill (Gaustec, Belo Horizonte, Brazil). The intensity in the exercise session represented approximately 95% of the mean lactate threshold for streptozotocin-induced T1DM rats, which was 19 m/min (unpublished results from our laboratory). All exercise sessions were performed in the morning, at 8 am. A non-exercise control situation replicated stress induced by oral gavage. Blood glucose levels were measured 60 minutes after oral gavage.

### Experimental design (second experiment)

Normoglycemic (NORM; 253.4 ± 7.3 g of body weight) and T2DM (255.9 ± 8.4 g of body weight) rats underwent two randomized experimental conditions: two incremental running tests after consumption of either CF or placebo supplementation. These sessions were spaced by at least 48 hours of resting during a washout period. The supplementation (CF or placebo) was administered to the animals 1 hour before the exercise session. Ten capillary blood glucose concentrations were measured at the following intervals: 60 minutes and 30 minutes before gavage, before exercise, immediately after exercise, and 3, 5, 10, 15, 30, and 60 minutes after exercise.

#### Type 2 diabetes mellitus induction and oral glucose tolerance test

We induced T2DM-related insulin resistance (T2DM, n = 10) using *ad libitum* high-fat diet and fructose-rich beverage for 30 days (^21^). This model promotes insulin resistance, increases inflammatory cytokines, and alters histomorphometric parameters in the liver, pancreas, and adipose tissue, which are characteristic of human insulin resistance (^21^). As controls, rats with normal glucose tolerance (NORM, n = 10) received a standard diet (Presence Nutrição Animal, Rio Verde, GO, Brazil) for 30 days. Glucose intolerance was confirmed by an oral glucose tolerance test (OGTT) after 6 hours of fasting, which is a well-established method in both clinical and basic research settings for detecting prediabetes and T2DM (^22^). Blood samples were obtained by lancing the distal tail at baseline and following oral gavage of a glucose solution (1 g/kg, 40% solution; Synth, Diadema, SP, Brazil) (^21^).

#### Incremental running test

The incremental running test started at a pace of 10 m/min, with the speed increasing by 1 m/min every 3 minutes. Fatigue was determined to have occurred when the rats remained on the electrical stimulation grid for 10 uninterrupted seconds (^20^). During the incremental test, oxygen uptake was measured continuously using open-flow indirect calorimetry (Gas Analyzer; Panlab, S.L./Harvard Apparatus Spain, Cornellà, Spain), and peak oxygen uptake (VO2peak) and external work were calculated. The external work was calculated in Joules as *bm × g × s × sin(Ø) × t*, where bm is the animal’s body mass (kg), g is the acceleration due to gravity (9.8 m/s^2^), *s* is the treadmill speed (m/min), θ is the angle of treadmill inclination, and t is the time spent at each stage (^23^). All tests were performed in the morning, at 8 am.

#### Placebo and cocoa flavanol supplementation

Flavanol-enriched cocoa powder and placebo solution, both dissolved in water (18 mg/mL) were equilibrated for caffeine and theobromine concentrations (details of bioactive compounds are shown in Table 1) (^23^). Oral gavage was used to administer CF and placebo supplementation (45 mg/kg body mass) (^23^). The same procedure was conducted in both experiments. Supplementation was performed by a blinded researcher.

**Table 1:** Concentration of bioactive compounds in the flavanol-rich cocoa powder extract (Naturex, Avignon, France) and placebo solution used in the study

Bioactive compounds	Naturex	Placebo
(+)-Catechin (g/100 g)	1.39	-
(-)-Epicatechin (g/100 g)	6.10	-
Caffeine (g/100 g)	0.89	0.89
Theobromine (g/100 g)	5.01	5.01

#### Capillary blood glucose assessment, exposure to hyperglycemia, and blood lactate analysis

Capillary blood glucose concentrations were measured using glucometer reagent strips (Accu-Chek Performa, Roche Diagnostics, France). Exposure to hyperglycemia was estimated by calculating the area under the curve (AUC) for capillary glucose.

Blood lactate concentrations were measured in blood samples (25 µL) collected from the same tail puncture used for capillary blood glucose assessments at baseline and immediately after the incremental running tests. The blood samples were placed in 60 µL of 1% sodium fluoride for later measurement of lactate concentrations, performed in duplicate using the electroenzymatic technique (YSI 1500 Sport, YSI Incorporated, Yellow Springs, OH, USA).

### Statistical analysis

The sample size was calculated using the software G*Power 3 (^24^), with an alpha level of 0.05 and power (1 - ß) of 0.80. Glucose concentrations from a pilot project were used to calculate the partial eta squared and effect size. For the first experiment - which included one group (T1DM) and three conditions (CON, CF, and PLA) - eta squared (η^2^ = 0.04) and effect size (0.20) were used, resulting in a total sample size of 8 rats per group and an actual power of 0.85. For the second experiment - which included two groups (NORM and T2DM) and two conditions (PLA, COCOA) - eta squared (η^2^ = 0.05) and effect size (0.22) were used, resulting in a total sample size of 10 rats per group and an actual power > 0.90.

Data normality and homoscedasticity were assessed using the Shapiro-Wilk test and Levene’s tests, respectively. The results are shown as mean ± standard deviation (SD) or standard error of the mean (SEM), except when noted in the figures. Capillary blood glucose concentrations over time (first experiment), capillary blood glucose in response to OGTT, effects of supplementation on aerobic parameters, and lactate concentrations (second experiment) were compared using two-way analysis of variance (ANOVA) with repeated measures. The hyperglycemic exposure was calculated based on the AUC using trapezoidal rule integration and compared using one-way ANOVA with repeated measures for the first experiment (group × supplementation) and two-way ANOVA with repeated measures for the second experiment (supplementation × placebo × control condition). Capillary blood glucose levels over time and lactate concentrations were compared using three-way ANOVA (second experiment). The AUCs of glucose in response to OGTT at both time points were compared using two-way ANOVA. *Post hoc* tests appropriate to the coefficient of variation were used to detect differences between means.

The statistical analyses were performed using the software SigmaPlot, version 11.0 (SYSTAT Software, Inc., Chicago, IL, USA). Graphs were created using GraphPad Prism, version 5.0 (GraphPad Software, Boston, MA, USA). The significance level was set at 5%.

## RESULTS

### First experiment

Capillary blood glucose concentrations were measured at the beginning of the light (7 am) and dark (7 pm) cycles. The glucose result was 378 ± 28 mg/dL (range 345-436 mg/dL), indicating that the T1DM rats remained hyperglycemic most of the day.

**Figure 1:**
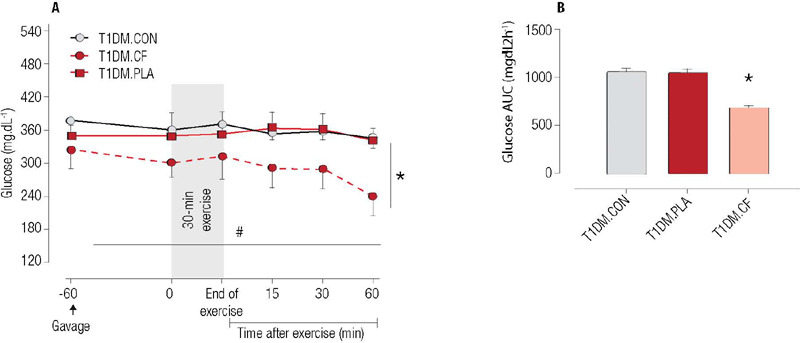
Acute effects of cocoa flavanol supplementation on glucose concentration before and after 30 minutes of aerobic exercise performed at continuous speed (n = 8). Data are presented as mean (± standard error of the mean). Note: Moderate-intensity aerobic exercise sessions post-consumption of either cocoa flavanol supplementation (T1 DM.FLA) or placebo (T1DM.PLA) and a non-exercise, non-supplementation condition (T1DM. CON). * P < 0.05: difference between conditions (FLA *versus* PLA and CON); # P < 0.05: difference over time.

The primary effect of CF supplementation on blood glucose occurred following an aerobic exercise session. A significant main effect of time was observed (*F* = 7.990; p = 0.006), indicating a reduction in glucose levels over time. Additionally, a condition effect (*F* = 4.009; p = 0.012) was observed (*F* = 4.009; p = 0.012), indicating that the CF condition differed from the PLA and CON conditions (Figure 1A). The CF supplementation led to a 23% reduction in capillary glucose levels 60 minutes after exercise, while PLA and CON reduced levels by only 1% and 3%, respectively. Additionally, CF supplementation resulted in lower glucose exposure compared with the PLA and CON conditions (*F* = 12.05; p = 0.001) (Figure 1B).

The changes in capillary blood glucose levels before and immediately after exercise in the CF, PLA, and CON groups were comparable (2%, -2%, and -3%, respectively; p > 0.05 for all). This indicates that exercise, compared to an absence of exercise (CON), did not significantly affect capillary blood glucose levels in T1DM rats, regardless of CF supplementation.

### Second experiment

**Figure 2:**
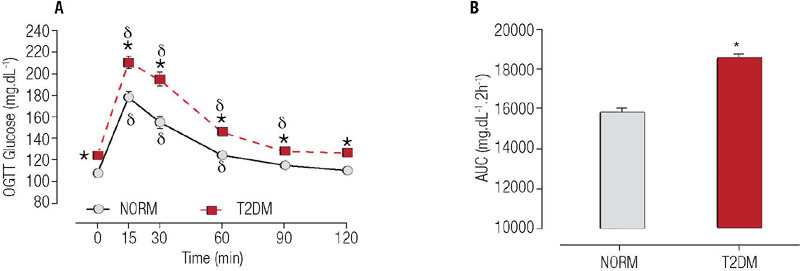
Oral glucose tolerance test 30 days after diet initiation. Data are presented as mean (± standard error of the mean). Note: NORM, normoglycemic rats (n = 10); T2DM, type 2 diabetes rats (n = 10). * P < 0.05, difference between groups. d indicates p < 0.05, difference from baseline (0). **Main effects by ANOVA:** Time *F* ( = 149.297, p < 0.001); Group (*F* = 46.675, p < 0.001); Group × Time (*F* = 4.872, p < 0.001). Area under the curve (AUC) of glucose: Tukey’s test (*t* = -5.622, p < 0.001).

Insulin resistance was confirmed by OGTT following 30 days of diet. Rats with T2DM, compared with NORM rats, exhibited higher glucose concentrations (Figure 2A-B) and greater body mass (401.37 ± 39.23 g *versus* 380.81 ± 27.61 g, respectively; p = 0.041).

Following the incremental running test, the T2DM rats exhibited hyperglycemia in the PLA condition compared with NORM rats under the same condition (Figure 4A). Interestingly, acute CF supplementation attenuated this hyperglycemic status after running in T2DM rats, as they maintained glycemia at levels similar to those of NORM rats supplemented with placebo (see Figure 2A-B). Moreover, glucose concentrations promptly returned to baseline levels 15 minutes after the incremental running test in T2DM rats supplemented with CF. Glucose concentrations also returned to baseline levels in NORM rats 10 minutes after the incremental running test when supplemented with CF. Thus, CF supplementation blunted the hyperglycemic response induced by aerobic exercise in both T2DM and NORM rats.

**Figure 3:**
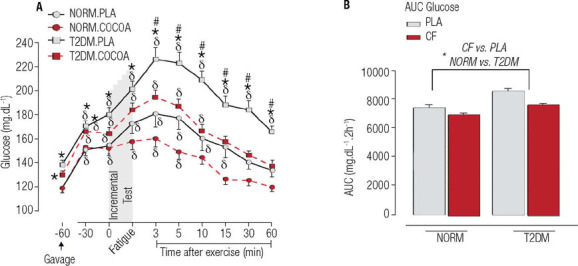
Acute effects of cocoa flavanol supplementation on glucose concentrations before and after the incremental running test. Data are presented as mean (± standard error of the mean). Abbreviations: NORM, normoglycemic rats (n = 10); T2DM, type 2 diabetes rats (n = 10). * P < 0.05, difference between groups (T2DM *versus* NORM). d indicates p < 0.05, difference from baseline; # indicates p < 0.05, difference from placebo. **Glucose, main effects by ANOVA:** Group *F* (= 327.480, p < 0.001); Supplementation (*F* = 91.624, p < 0.001); Time (*F* = 40.929, p < 0.001); Group × Supplementation (*F* = 14.589, *p* < 0.001); Group × Time (*F* = 3.081, p = 0.001); Supplementation × Time (*F* = 4.035; p < 0.001). Area under the curve (AUC), main effects **by ANOVA:** Group *F* (= 28.638, p < 0.001); Supplementation (*F* = 20.302, p < 0.001).

When comparing T2DM and NORM rats, regardless of supplementation type, there were no differences in VO_2_peak, velocity peak (Vpeak), or total external work (Figure 3A,B,C). Acute CF supplementation did not influence aerobic parameters (VO_2_peak, Vpeak, or total external work). Lactate concentration increased after the incremental running test in both groups. However, T2DM rats exhibited a higher increase than NORM rats, regardless of CF supplementation (Figure 3D). Thus, CF supplementation did not affect aerobic performance parameters or lactate response in T2DM or NORM rats, although the lactate response differed between T2DM and NORM rats.

**Figure 4:**
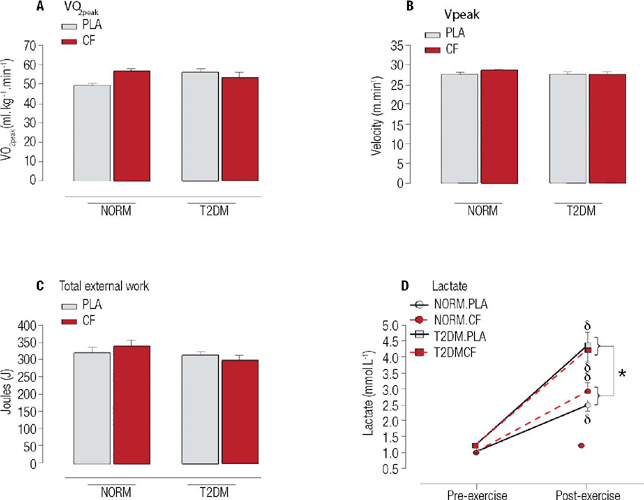
Acute effects of cocoa flavanol supplementation on physical performance parameters and lactate concentrations. Data are presented as mean (± standard error of the mean). Abbreviations: NORM, normoglycemic rats (n = 10); T2DM, type 2 diabetes rats (n = 10); VO2peak, oxygen uptake peak; Vpeak, velocity peak. * P < 0.05, difference between groups. indicates p < 0.05, difference from pre-exercise. Lactate, main effects by ANOVA: Moment (*F* = 160.093, p < 0.001), Group (*F* = 44.204, p < 0.001), Group x Moment (*F* = 8.468, p = 0.004).

## DISCUSSION

Our study revealed the novel finding that acute CF supplementation prior to aerobic exercise enhances glycemic reduction, effectively reducing the time of exposure to hyperglycemia, regardless of the glucometabolic condition. Notably, CF supplementation did not influence aerobic performance or lactate concentration after a high-intensity incremental running test in T2DM rats.

Supplementing CF before aerobic exercise led to a significant reduction in capillary blood glucose within the first hour after exercise. Notably, bioactive cocoa monomers, such as +(-)catechin and -(-)epicatechin, remain detectable in rat and human plasma for up to 6 to 8 hours following CF supplementation, with detectable levels at, respectively, 60 and 90 minutes post-supplementation (^25,26^). Since, in the present study, the exercises started at the peak of these bioactive cocoa monomers in plasma, their effects on glucose uptake likely contributed to the observed enhancements during exercise recovery.

Numerous physiological mechanisms underlie the benefits of CF on glucose dynamics. Existing literature shows that CF regulates glucose transport and critical proteins in the insulin signaling pathway (IR, IRS1, AKT, ERK1/2, and GLUT-4 translocation) (^27^). The hypoglycemic effects of cocoa may involve the modulation of enzymes such as phosphoenolpyruvate carboxykinase and glucokinase, which are key players in gluconeogenesis and regulation of carbohydrate metabolism (^27^). Additionally, CF-rich diets have been linked to reduced activation of c-Jun N-terminal kinase (JNK) and p38, which are involved with the inflammatory insulin resistance phenotype (^16,27^). Indeed, blood glucose concentration is regulated by glucose uptake and disposal, and CF increases nitric oxide bioavailability and induces peripheral vasodilation, (^28^) further contributing to glucose uptake in peripheral tissues (^29^). Despite these positive effects, CF may attenuate glycolysis and gluconeogenesis after exercise, influencing glucose disposal (^16,27,30^) and potentially contributing to glycemic reduction after aerobic exercise.

In the first experiment of the present study, we investigated three distinct metabolic conditions in rats with streptozotocin-induced T1DM, characterized by pancreatic beta-cell death and dysfunctional glucose metabolism (^31^). The second experiment simulated a condition of T2DM-related insulin resistance induced by a high-fat diet and fructose-rich beverage, representing prediabetes and early T2DM. Normoglycemic animals were included to assess the impact of CF on glycemic reduction in the absence of disrupted insulin metabolism. Compared with placebo, CF induced higher glycemic reduction in T1DM rats (10.0-fold), T2DM rats (2.0-fold), and normoglycemic rats (1.5-fold) 60 minutes after aerobic exercise.

Notably, higher pre-exercise blood glucose levels correlate with higher glycemic reductions immediately after aerobic exercise (^32^). However, the intensity of the aerobic exercise (moderate *versus* vigorous) may also modulate glycemic reduction (^4,33^), a finding that warrants further investigation. While studies have explored CF supplementation in athletes without diabetes (^34,35^), its effects on physical performance among individuals with insulin resistance have not been investigated yet. Our findings align with a systematic review showing that acute CF intake does not significantly improve exercise performance in adults across various weight statuses (^35^).

Aerobic parameters (VO2peak, Vpeak, and total external work) remained similar between placebo and CF supplementation in normoglycemic and insulin-resistant rats. While studies have explored CF supplementation in nondiabetic athletes (^34,35^), its effects on physical performance in insulin-resistant populations have not been investigated. During exercise, CF can induce several physiological and metabolic changes, such as improvements in vascular function, reductions in exercise-induced oxidative stress, and alterations in lipids and carbohydrate utilization (^35^). The findings of the present study are aligned with a systematic review showing that acute CF intake does not significantly improve exercise performance in adults across various weight statuses (^35^). They also reinforce the evidence that a single CF administration may be insufficient to increase aerobic performance in normoglycemic and insulin-resistant rats.

As expected, lactate concentrations increased after the incremental running tests. The T2DM rats exhibited higher post-exercise lactate values compared with the rats in the CON group, suggesting an alteration in oxidative capacity at a cellular level, potentially contributing to insulin resistance-related abnormalities in lactate concentrations following aerobic exercise (^36^). Reductions in mitochondrial size and density (^37^), along with attenuated expression of oxidative genes (^38^), contribute to impairments in oxidative phosphorylation and aerobic capacity (^39^). These factors may explain the observed abnormal increase in blood lactate concentration following aerobic exercise. Given that lactate concentrations during physical exercise are determined by a balance between production and removal, it is tempting to speculate that an inefficient buffering or impaired removal system may also contribute to higher lactate accumulation in conditions such as T2DM.

Aerobic exercise enhances glucose absorption, resulting in a tenfold increase in glucose uptake in active leg muscle fibers during low-intensity aerobic exercise (^40^). Different exercise regimens can induce different glycemic responses. For instance, while low-intensity exercise can reduce blood glucose levels, high-intensity exercise may increase glucose levels in individuals with T1DM (^4^), but not necessarily in those with T2DM (^41^).

Due to insulin deficiency, exogenous insulin is necessary to maintain blood glucose within an appropriate range in T1DM. It is important to note that exogenous insulin remains active during exercise, unlike endogenous insulin, which has reduced secretion during physical activity (^40^). This sustained insulin activity at elevated rates may lead to increased glucose uptake, potentially resulting in hypoglycemia (^4^), a situation that may not occur in individuals with T2DM (^41^). In our study, T1DM rats received two daily insulin doses, but the morning insulin dose was withheld on experiment days to prevent hypoglycemic events that could compromise the study. This insulin withdrawal induced pre-exercise hyperglycemia in T1DM rats, which reached values higher than 300 mg/dL, in contrast to the values of 130-140 mg/dL and 120-125 mg/dL found in T2DM and normoglycemic rats, respectively. Consequently, a moderate-intensity aerobic exercise protocol below the anaerobic threshold was chosen for T1DM rats to avoid exacerbating their already hyperglycemic status through counterregulatory responses associated with high-intensity incremental aerobic exercise used in the second experiment. While aerobic exercises with moderate and vigorous intensity are widely recommended for individuals with T2DM (^3,42,43^), the increase in glycogenolysis and hepatic gluconeogenesis resulting from vigorous-intensity aerobic exercises can induce temporary hyperglycemia after the exercise (^33^).

The results of our study also showed that acute CF supplementation prior to high-intensity physical exercise efficiently mitigated post-exercise hyperglycemia in T2DM rats. Although the precise cellular mechanisms remain to be elucidated, the ongoing ECODIA study, a collaborative effort across institutions (UFMG, VUB, and Université de Lille), aims to investigate the combined effects of physical exercise and cocoa supplementation on diabetes-induced metabolic dysfunction, with controlled randomized trials in humans and mechanistic experiments in rats.

The present study has several strengths. Using animal models with distinct glucometabolic conditions (rats with insulin deficiency or T1DM, insulin resistance or T2DM, and normoglycemia) to investigate the effect of CF combined with aerobic exercise on capillary glucose, along with the observation of these glycemic responses 60 minutes after the end of the exercise (up to 150 minutes after oral gavage) provides insights into the clinical applicability of the results in the real world. However, this study also has limitations, such as the absence of investigation of physiological mechanisms associated with glucose uptake or gluconeogenesis. We previously demonstrated that a combination of aerobic training and CF are effective therapies to reduce metabolic and inflammatory disruptions in insulin-resistant rats (^23^). Thus, future studies can explore the physiological mechanisms underlying the glucose uptake responses mediated by CF and physical exercise.

In conclusion, the findings of our study show practical effectiveness in CF supplementation, potentiating capillary blood glucose reduction following aerobic exercise and minimizing exposure to high glycemic values in conditions of insulin deficiency, obesity-related insulin resistance, and normoglycemia. Future investigations should explore the long-term effects of CF consumption on glycemic control markers.

In conclusion, supplementation with CF before physical exercise potentiates glycemic reduction following aerobic exercise by attenuating a hyperglycemia-induced status after an incremental running test. Therefore, CF supplementation may be an interesting strategy for reducing blood glucose levels when combined with aerobic exercise, regardless of glucometabolic condition.
